# Episodic memory improvement in community-dwelling women following a remote language-based stimulation program

**DOI:** 10.1590/1980-5764-DN-2024-0248

**Published:** 2025-07-11

**Authors:** Vanessa Bisol, Bárbara Luzia Covatti Malcorra, Bárbara Rusch da Rocha, Ana Luiza Licodiedoff Peruzzo, Luciana Zanatta, Letícia Priscila Pacheco, Erica dos Santos Rodrigues, Maria Teresa Carthery-Goulart, Lilian Cristine Hübner

**Affiliations:** 1 Pontifícia Universidade Católica do Rio Grande do Sul, Escola de Ciências da Saúde e da Vida, Curso de Psicologia, Porto Alegre RS, Brazil.; 2 Pontifícia Universidade Católica do Rio Grande do Sul, Escola de Humanidades, Programa de Pós-Graduação em Letras-Linguística, Porto Alegre RS, Brazil.; 3 Pontifícia Universidade Católica do Rio de Janeiro, Rio de Janeiro RJ, Brazil.; 4 Conselho Nacional de Desenvolvimento Científico e Tecnológico, Brasília DF, Brazil.; 5 Universidade Federal do ABC, Centro de Matemática, Computação e Cognição, São Paulo SP, Brazil.; 6 Universidade de São Paulo, Faculdade de Medicina, Departamento de Neurologia, Grupo de Neurologia Cognitiva e do Comportamento, São Paulo SP, Brazil.; 7 University of Hong Kong, Faculty of Education, Human Communication, Learning, and Development Unit, Hong Kong-SAR.; 8 Instituto Federal Sul-Rio-Grandense, Venâncio Aires RS, Brazil.

**Keywords:** Memory, Episodic, Language Training, Cognitive Training, Telehealth, Aging, Cognitive Dysfunction, Memória Episódica, Terapia da Linguagem, Treino Cognitivo, Telessaúde, Envelhecimento, Disfunção Cognitiva

## Abstract

The increasing aging of the world's population has motivated studies leading to initiatives like developing online and face-to-face cognitive stimulation programs targeting typical and atypical aging populations. The decline in episodic memory (EM) capacity is one of the hallmarks of cognitive decline in Alzheimer's disease.

**Objective:**

The study analyzes the effect of a telepractice, composed exclusively of language activities, on five tasks of EM by comparing their scores in pre- and post-intervention assessments.

**Methods:**

Forty-nine (49) women aged 57–83 years (mean 68.1), with 6–22 (mean 15.1) years of formal education, engaged in a 15-session online intervention program delivered daily during the COVID-19 pandemic. A pre- and post-intervention cognitive assessment was administered, including five tasks assessing EM: two subtests of the verbal learning task of the Battery for the Assessment of Language in Aging (BALE) (free recall and with cues), the delayed recall subtest of the Addenbrooke's Cognitive Examination-Revised (ACE-R), the recall of the Brief Cognitive Screening Battery (BBRC) and the Face and Name Recall Test.

**Results:**

EM scores were consistently higher in the post-intervention assessments, with a significant improvement observed in four of the five EM tasks.

**Conclusion:**

The results bring implications for further research about telepractice, suggesting that typical older adults can benefit from language-based cognitive stimulation to prevent, reduce, or rehabilitate EM deficits.

## RESUMO.

O envelhecimento crescente da população mundial tem motivado estudos que levam a iniciativas como o desenvolvimento de programas de estimulação cognitiva *online* e presenciais, direcionados a populações com envelhecimento típico e atípico. O declínio da capacidade da memória episódica (ME) é uma das principais características do declínio cognitivo na doença de Alzheimer.

**Objetivo:**

Este estudo analisa o efeito de uma teleprática, composta exclusivamente de atividades de linguagem, em cinco tarefas de ME, comparando seus escores nas avaliações pré e pós-intervenção.

**Métodos:**

Quarenta e nove mulheres com idades entre 57 e 83 anos (média de 68,1) e com 6 a 22 anos (média de 15,1) de escolaridade participaram de um programa de intervenção *online* com 15 sessões, realizado diariamente durante a pandemia da COVID-19. Foi realizada uma avaliação cognitiva antes e depois da intervenção, incluindo cinco tarefas para avaliar a ME: dois subtestes da tarefa de aprendizagem verbal da Bateria de Avaliação da Linguagem no Envelhecimento (BALE) (recordação livre e com pistas), o subteste de recordação tardia do Exame Cognitivo de Addenbrooke-Revisado (ACE-R), a recordação da Bateria Breve de Rastreio Cognitivo (BBRC) e o Teste de Recordação de Rostos e Nomes.

**Resultados:**

Os escores de ME foram consistentemente mais altos nas avaliações pós-intervenção, com melhora significativa observada em quatro das cinco tarefas de ME.

**Conclusão:**

Os resultados trazem implicações para futuras pesquisas sobre teleprática, sugerindo que adultos mais velhos típicos podem se beneficiar da estimulação cognitiva baseada em linguagem para prevenir, reduzir ou reabilitar déficits de ME.

## INTRODUCTION

The world's population aged 60 or over is projected to more than double, surpassing one-third of the world population by the end of the century^1^. Advancing age increases the susceptibility to neurodegenerative pathologies, affecting the clinical sphere and the social and economic agendas.

Long-term and working memories decline with age^2^. However, language-related skills tend to remain stable, mainly due to the accumulation of vocabulary and the continuous improvement of the linguistic repertoire throughout life^3^. Decreased EM makes learning and retrieving recent personal events progressively more difficult^4^ and is a hallmark of Alzheimer's disease (AD)^5^.

Rehabilitation, training, and stimulation interventions have emerged as practical tools to preserve or enhance cognitive abilities in adults and older adults. These approaches contribute to a better quality of life, greater functionality, and increased autonomy in daily activities^3^. This occurs because the aging brain retains a remarkable ability to adjust and reorganize itself through cognitive engagement activities, which delays age-related cognitive decline and reduces the risk of developing AD symptoms^6^. Positive changes in neural activity and brain structure can occur in response to cognitive training, which indicates cognitive plasticity^7^.

Social isolation has been considered a risk factor that significantly intensifies both subjective and clinical cognitive decline, particularly among high-risk older adults^8^. During the COVID-19 pandemic, stringent social distancing measures disrupted daily routines, leading to reduced cognitive and social engagement opportunities. This, in turn, exacerbated social isolation among older adults. Nevertheless, research has shown that cognitive training can mitigate these effects by improving cognitive function in older adults, even under conditions of restricted social interaction. This is achieved through enhancements in functional brain connectivity and the activation of compensatory mechanisms that help counteract the cognitive decline associated with aging and neurodegenerative conditions^9^. Thus, training can be delivered face-to-face or in a telepractice, the latter being reinforced during the COVID-19 pandemic as a promising healthcare opportunity, offering flexibility and access to specialized services^10,11^. However, challenges such as lower supervision and social interaction in home-based interventions exist, highlighting the need to evaluate hybrid models combining center-based and home-based sessions^12^ and implementing supervision and feedback practices into the remote models for better adherence and motivation^13^.

Scientific evidence supports the effectiveness of training in improving cognitive abilities and preventing cognitive decline in healthy older adults. For instance, the study by Irigaray et al.^14^ demonstrated positive effects on executive functions post-intervention in a sample of 76 older adults aged 60 to 89. Additionally, the study^15^ examined 39 studies in a systematic review and meta-analysis, concluding that cognitive stimulation and training can enhance cognitive functions and emotional well-being. Multi-domain programs, in particular, showed more pronounced improvements in white matter integrity, cognitive processing speed, and induced neural plasticity changes in the frontal cortex compared to single-domain programs. According to the authors^16^, these practices not only promote neuroplasticity but also help delay the onset of neurodegenerative diseases, serving as a crucial preventive measure for maintaining long-term cognitive health in community-dwelling older adults.

Based on the assumption that language remains relatively stable with aging, a linguistic-cognitive telepractice was developed, consisting exclusively of language tasks at the word, sentence, and text levels. The multicentric umbrella program was administered during COVID-19 to participants all over the Brazilian territory. In the present study, we examined the immediate effects of the program on EM measures in a sample consisting exclusively of older community-dwelling women who completed all phases of the program. To our knowledge, no previous study has investigated the effects of an exclusively language-based program on EM. The rationale for proposing a language-based cognitive stimulation program is based on the idea that distinct cognitive resources are utilized in language processing^10,11^. As a result, these tasks can indirectly enhance executive functions and different memory systems.

## METHODS

The university ethics committee approved the study under Certificate of Presentation for Ethical Appreciation (CAEE) number 53696221.4.1001.5336, and participants signed an informed consent form.

### Participants

Forty-nine older adult women participated in the study (Table 1 for their sociodemographic and neuropsychological characteristics). As an inclusion criterion, to control for the expected cognitive differences related to sex/gender mentioned in the literature^17^, only women were included in the study. On the other hand, data from younger adults (aged 49 or under) and individuals with a history of active neurological or psychiatric illnesses were excluded from the analysis. Participants who had not completed all of the telepractice activities were also excluded. A series of psychoeducational lectures on health in aging, featuring guests from various specialties (nutritionists, speech therapists, physicians, psychologists, neuroscientists), was offered over Zoom during the recruitment period. The researchers hosted the sessions and promoted the study, explaining its main goals and procedures, and inviting those interested in participating to enroll via a Google Form. All participants had a general cognitive performance within the normal range as measured by the Mini-Mental State Examination^18^. We followed the Brazilian scoring procedure provided by Laks et al.^19^, which was adapted for the Brazilian population and considered age and education level. Participants were mostly of upper-middle socioeconomic status (SES)^20^. A questionnaire assessed reading and writing habits (RWH) and the history of literacy development^21^.

**Table 1:** Descriptive analyses.

Variables	Mean	SD	Range
Age	68.1	6.08	57–83
Education (in years)	15.1	3.7	6–22
Reading habits	53.8	31.3	0–100
Writing habits	32.6	30.9	0–100
SES	38.7	13.4	10–72
MMSE	28.2	1.3	25–30

Abbreviations: SD, standard deviation; MMSE, Mini-Mental State Exam; SES, socioeconomic status; RWH, reading and writing habits.

Notes: MMSE with cut-off points established by Laks et al.^19^; SES^18^ (lower = 0–16; middle = 17–28; upper middle = 29–44; upper = 45–100); RWH and the history of literacy and learning development (maximum score: 124; lower scores indicate higher RWH)^19^.

### Data collection instruments and procedures

A multicentric and multidisciplinary group of Brazilian researchers developed the linguistic-cognitive stimulation and training program during the COVID-19 pandemic^10,11^. It was implemented on the Moodle platform.

After recruitment, participants received consent forms and underwent a synchronous individual assessment. They answered questionnaires on sociodemographic and clinical data. They were also evaluated using cognitive measures adapted for online administration via Zoom. It is essential to highlight that the test content remained unchanged, and the application process was carefully designed to replicate face-to-face assessments as closely as possible. The adaptation was minimal, with changes made only to the assessment format when necessary to ensure a smooth remote application. This included presenting stimuli through shared PowerPoint or Word files on screen, instead of the traditional paper-based format used in face-to-face assessments. Additionally, participants were requested to have paper and a pencil or pen ready for tasks involving writing or drawing. The drawing and writing outputs were displayed to the examiner online and also submitted as a photo to the examiner. All assessments were recorded via video and uploaded to a password-protected drive, as previously explained when the participants signed the Informed Consent Form. After recruitment, participants were added to Moodle, a personalized virtual learning environment, configured to include links, files, and forums. The telepractice lasted 15 days (three weeks). Participants performed daily tasks lasting approximately 20 minutes five times a week. The linguistic and gamified activities involved reasoning, reading, writing, vocabulary, syntactic structures, and text comprehension. Each activity day had a learning block with four tasks, released automatically at midnight on the scheduled day. This configuration prevented participants from anticipating the activities. The team kept track of each participant's login time and the number of attempts and errors in the activities. Each participant received individual technical support from a tutor available via WhatsApp. After the end of the program, the participants were reassessed.

#### Episodic memory tasks

Four instruments assessed EM:

- Two tasks of the verbal learning task from the Battery for Language Assessment in Aging^22^ (identifying and learning 16 figures from different semantic categories of nouns, grouped in four charts with four items each, with three recalls: two times free immediate recall plus cued immediate recall and a 20-min delayed recall. The scores of the delayed free and cued recall were analyzed);
- Brief Cognitive Screening Battery (BBRC)^23^ (ten drawn figures that the participants identified, named, and were prompted to learn them for later recall; after 5 minutes, EM is assessed by recalling the figures again, and performing a recognition task);
- ACE-R^24^ (memory task: the participant is instructed to memorize a name and address, which is repeated three times in the learning phase [immediate memory]; at the end of the test [around 5 minutes later], they are asked to recall the name and address and are given cues in case they fail in the free recall phase);
- Face and Name learning task^25^ (ten faces paired with ten names [first and last names]; the test includes a learning phase [three attempts], 5-minute delayed free recall, and recognition). The test has two versions (A and B) used in the pre-and post-test assessment. Each subject was randomly assigned to receive the A or B version first.

### Statistical analysis

We used the Shapiro-Wilk test to check whether the difference between pre-stimulation and post-stimulation was normally distributed for the five variables of interest. Since the difference was not normally distributed (p<0.05), we used the non-parametric paired t-test (Wilcoxon test) to compare pre-stimulation and post-stimulation performances. Correction for multiple comparisons using the Bonferroni method included five comparisons (correct α=0.0125) in four tasks administered. All the analyses were performed in RStudio 4.1.0^26^.

## RESULTS

Table 1 provides the means, standard deviations, and ranges (minimum and maximum values) for the sample's sociodemographic and neuropsychological variables.

**Figure 1:**
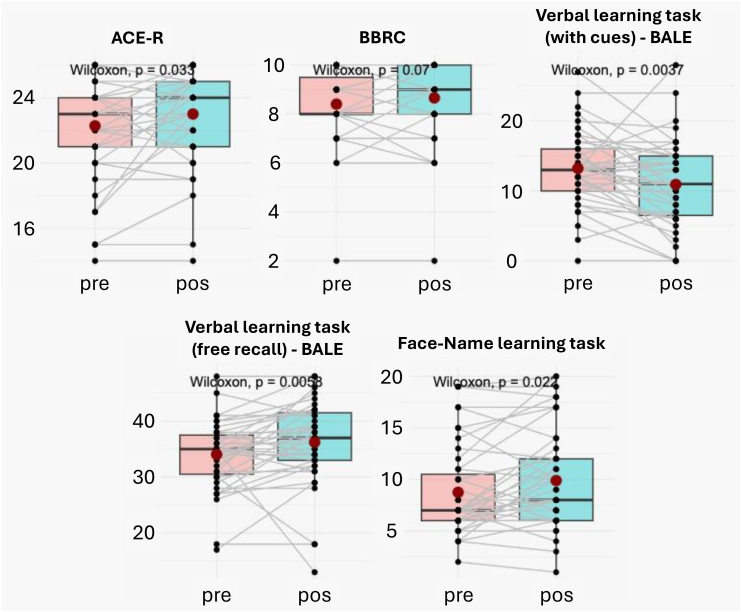
Box plots comparing pre-stimulation and post-stimulation.

Note: The red dots on the box plots represent the average of the values.

Significant differences were observed between pre-stimulation and post-stimulation on the verbal learning task (with cues) (V=108.5, p=0.004), verbal learning task (free recall) (V=411.5, p=0.006), faces and names recall (V=454.5, p=0.022) and ACE-R (V=224.5, p=0.033), but not on the delayed recall subtest of the BBRC (V=211.5, p=0.070) (Table 2). The post-stimulation averages are higher than the pre-stimulation average on all the variables, except for verbal learning tasks (with cues), in which the post-stimulation averages are lower than the pre-stimulation (Figure 1).

**Table 2:** Comparison between pre-stimulation and post-stimulation.

Variables	Pre-stimulation		Post-stimulation		p-value
	Mean	SD	Mean	SD	
Verbal learning task (with cues) - BALE	13.23	5.30	10.93	6.16	108.5 **0.004**
Verbal learning task (free recall) - BALE	34.05	6.04	36.23	7.62	411.5 **0.006**
Face-Name learning task	8.74	4.35	9.89	4.72	454.5 **0.022**
ACE-R	22.28	3.12	23.00	2.85	224.5 **0.033**
BBRC	8.40	1.48	8.66	1.54	211.5 0.070

Abbreviations: SD, standard deviation; BALE, Battery of Language Assessment in Aging^20^; ACE-R, Addenbrooke's Cognitive Examination - Revised^22^; BBRC, Brief Cognitive Screening Battery^21^.

Note: Bold values are less than 0.05.

## DISCUSSION

The decline in EM capacity is a hallmark of AD^5^, and cognitive stimulation can help reduce the deterioration of this function associated with aging. In a relatively short online language-based program (15-day intervention), the participants improved their EM ability in four out of five tasks used to measure this construct.

Importantly, in our study, this effect was observed despite the short duration of the intervention. This suggests that even short interventions may positively impact EM, an essential cognitive construct to cope with the cognitive challenges faced by the elderly population. Furthermore, this improvement was obtained through stimulation activities tapping on vocabulary exploration, syntactic construction analyses, and text-based tasks, including reading comprehension and making inferences. This shows the efficiency of language-related tasks in improving other cognitively related constructs, such as EM in this case.

Although the improvement in the BBRC delayed-recall test was not statistically significant, it demonstrated the same trend as the other EM measures: participants were able to freely recall more items in the post-intervention assessment and benefited from cues, as observed in the cued-recall task of BALE. This finding is consistent with the nature of EM difficulties in typical aging, where memory storage remains relatively preserved, and retrieval difficulties are the primary cause of memory errors^27^.

The linguistic-cognitive stimulation and training program improved the participants’ EM ability, especially in tasks requiring delayed retrieval and recall of information. This is similar to the findings reported by the reviews of Mendonça et al.^7^ and Kaspary et al.^28^. Mendonça et al.^7^ analyzed 23 studies focused on EM training in healthy older adults and clinical populations and showed that the reported cognitive training interventions effectively mitigated EM decline, with a variation in efficacy depending on the characteristics of the clinical groups, training duration, mode of EM training, and type of control sample. Furthermore, EM training resulted in social and psychological well-being.

The participants in our study had 15 years of education on average, which is relatively high for the older Brazilian population, and demonstrated average reading habits and low writing habits throughout their lives. Reading habits have been shown to impact speech connectedness more than age and even schooling^29^. Its impact on cognitive constructs, such as EM, should be further investigated, especially in aging research in countries with low and middle-low education and SES, such as Brazil.

The study has some limitations, which may prevent the generalization of the results and require further investigation. First, the small sample size (49 participants) limits the generalizability of the results to a larger population. Second, this study investigated the impact of an intervention program in adults and older adults with upper-middle SES and average education levels, which does not allow the generalization of the results to illiterate or low education levels and very low SES. Third, the lack of a control group and the difficulty in eliminating potential learning effects on the cognitive assessments underscore the need for a careful interpretation of the results. Our findings provide evidence that the research question addressed in this study merits further exploration in a larger randomized controlled trial, considering the associated higher costs of such a study. Additionally, future research could incorporate a follow-up evaluation to determine if the enhancements noted in EM are maintained over an extended period, explore different intervention durations, and contrast in-person and online interventions.

Despite these limitations, it is worth highlighting some of the study's contributions, among them its online design with interactive and personalized resources, which was able to meet participants’ adherence and was influential in administering pre- and post-assessment and delivering the intervention, despite the issues posed by remote access to the average aging population, mainly in countries like Brazil. Telepractice like the one reported has a high potential for practical applications in research and clinics.

Interventions of this nature have a significant impact on both social and academic spheres. From a social perspective, they enhance quality of life, reduce social isolation, foster participant engagement, promote socio-emotional well-being, and help lower government expenditures associated with dementia treatment. Academically, these interventions are notable for their effectiveness in building cognitive reserve, which plays a crucial role in reducing the risk of dementia progression. Moreover, they contribute to the advancement of scientific knowledge by providing valuable insights into strategies for preventing and treating cognitive decline^30^.

Linguistic-cognitive interventions in healthy aging populations, including reading and writing stimulation, could become an effective strategy for improving cognition and preventing age-related cognitive decline. Providing environments that encourage cognitive engagement can significantly enhance the brain's capacity for adaptation, making cognitive stimulation a crucial factor in neurotypical cognitive aging.
